# GSH-Related Enzymes GPx4, Chac1, and GSTs and Redox Regulation of Ferroptosis in Cancer

**DOI:** 10.3390/ijms27146353

**Published:** 2026-07-17

**Authors:** Elena Kalinina

**Affiliations:** T.T. Berezov Department of Biochemistry, Peoples’ Friendship University of Russia (RUDN University), 6 Miklukho-Maklaya Street, 117198 Moscow, Russia; kalinina-ev@rudn.ru

**Keywords:** GSH, GPx4, Chac1, GSTs, ferroptosis, redox regulation, cancer cells

## Abstract

The tripeptide glutathione (GSH) is the most abundant cellular non-enzymatic antioxidant. The GSH system plays a crucial role in antioxidant defense against oxidative stress and in supporting cellular redox homeostasis, regulating the reduction of lipid peroxides, and protecting cells from ferroptosis depending on the GSH level, which is maintained in a state of dynamic equilibrium not only by the activities of GSH synthesis enzymes, transporters of GSH precursor amino acids, and GSH transporters, but also by the actions of GSH-related enzymes. Some GSH-related enzymes are key enzymes with antioxidant functions such as glutathione peroxidases (GPxs), especially GPx4, and glutathione S-transferases (GSTs), which use GSH as a co-substrate for the reduction of hydroperoxides to alcohols, whereas glutathione-specific gamma-glutamyl cyclotransferase 1 (ChaC1) degrades intracellular GSH, so they can correspondingly lead to suppression or induction of ferroptosis. Ferroptosis is characterized by a buildup of lipid peroxides due to excessive lipid peroxidation and iron accumulation, which results from redox imbalance between ferroptosis’s drivers and defense systems, including impaired cellular antioxidant systems, particularly disruptions of GSH metabolism. It appears pertinent to assess the influence on ferroptosis regulation by GSH-dependent enzymes that utilize the GSH pool in diverse ways. This review offers an updated exploration of the roles of GPx4, ChaC1, and GSTs in redox regulation of ferroptosis in cancer cells, with a focus on both the regulation of each enzyme’s activity and their possible interactions, considering the impact on the risk of ferroptosis induction.

## 1. Introduction

Glutathione (L-γ-glutamyl-L-cysteinyl-glycine, GSH) is synthesized at high concentrations (1–5 mM) and primarily exists in its reduced form (GSH), constituting over 98% of total glutathione under normal conditions [1,2]. The ratio between reduced (GSH) and oxidized glutathione (GSSG)—GSH/GSSG—is maintained by the activity of glutathione reductase (GR), which ensures that the 2GSH/GSSG and NADP/NADPH redox couples are in thermodynamic equilibrium, playing important roles in supporting cellular redox potential [3]. The elevated GSH levels in tumor cells are associated with tumor progression and increased resistance to chemotherapeutic drugs [4,5,6,7].

Cancer cells exhibit aberrant redox homeostasis [8]. Redox balance is disrupted, leading to enhanced reactive oxygen species (ROS) production due to the actions of several factors, including activation of oncogenes, aerobic glycolysis, and hypoxia; some anticancer agents (anthracyclines, alkylating agents, and platinum coordination complexes) also act by increasing ROS production [9]. As a consequence, hyperproliferation of tumor cells is accompanied by high ROS production, which is adaptively balanced with increasing cellular antioxidant status to optimize ROS-driven proliferation [8,10]. The central role of GSH in protecting cells from ROS is based on antioxidant enzyme-catalyzed reactions, efficient recycling pathways, and high intracellular GSH concentrations [11]. GSH directly scavenges ROS (hydrogen peroxide, superoxide, and peroxynitrite) [12,13,14] and serves as a redox cofactor for key enzymes with antioxidant functions such as glutathione peroxidases (GPxs), glutathione S-transferases (GSTs), and glutaredoxins (Grxs) [15]. GPxs and GSTs reduce hydroperoxides to alcohols and effectively defend against increased oxidative stress, supporting cellular redox homeostasis and limiting ROS to a tumor-promoting level [16], whereas Grxs are the other key players in the maintenance of cellular redox homeostasis through reduction of disulfides and deglutathionylation of proteins [17]. GSH plays a critical role not only in maintaining cellular redox homeostasis and redox-dependent regulation of cell cycle regulation, apoptosis, and immunological defense, but also in the metabolism of endogenous compounds (e.g., estrogens, leukotrienes, and prostaglandins) [18].

Ferroptosis, as an iron-dependent, non-apoptotic form of regulated cell death, acts as an innate tumor suppressor mechanism and participates in the biological processes of tumors [19]. Mesenchymal and dedifferentiated cancer cells are highly vulnerable to ferroptosis, as are tumor cells with resistance to chemotherapeutic agents that are usually unaffected by traditional therapies [20,21]. It has been suggested that ferroptosis is an innate tumor-suppressive mechanism; this is supported by the capacity of tumor suppressors to execute part of their tumor-suppression function depending on ferroptosis induction [19,22,23]. Currently, the induction of ferroptosis is an effective pathway in the development of more targeted tumor treatments (liver, gastrointestinal, pancreatic ductal adenocarcinomas, renal cancers, etc.), which include triggering ferroptosis in cancer with inducers while protecting healthy tissues from ferroptosis-related damage using inhibitors [22,24,25].

Ferroptosis is characterized by a buildup of lipid peroxides due to excessive lipid peroxidation (LPO) and iron accumulation resulting from redox imbalance between ferroptosis’s drivers and defense systems, including impaired cellular antioxidant systems, particularly disruptions of GSH metabolism [22,26,27]. The main defense mechanisms against ferroptosis have long been known, including ferroptosis suppressor protein 1 (FSP1) and the coenzyme Q10 (CoQ10)–NAD(P)H/FSP1/CoQ10 axis [28]; GTP cyclohydrolase 1 and the tetrahydrobiopterin–GCH1/BH4 axis [29,30]; dihydroorotate dehydrogenase and the ubiquinol–DHODH/CoQH2 axis [31]; and the system Xc−/GSH/GPx4 pathway, including the cystine–glutamate antiporter Xc− needed for cysteine transport and GSH synthesis; as well glutathione peroxidase 4 (GPx4), which reduces lipid hydroperoxides using as GSH as a co-substrate [22,32,33]. However, many questions regarding its regulation still remain.

The GSH system plays a crucial role in antioxidant defense against ROS, regulating the reduction of lipid peroxides and protecting cells from ferroptosis depending on the GSH level, which is maintained in a state of dynamic equilibrium through the intricate regulation of its synthesis, utilization, distribution, uptake, and decomposition [34]. Targeting GSH is the core link and regulatory switch to induce ferroptosis [35].

GSH is consumed by enzymes involved in antioxidant defense, detoxification, and cellular metabolism, including GPxs, GSTs, and Grx, serving in antioxidant protection, whereas gamma-glutamyl transpeptidase (GGT) is used for extracellular GSH and GSSG degradation and cysteine uptake for intracellular GSH resynthesis, while glutathione-specific gamma-glutamyl cyclotransferases (ChaC1/ChaC2) catalyze intracellular GSH degradation, which can lead to induction of ferroptosis, especially during excessive endoplasmic reticulum stress [36,37,38]. These enzymes affect cellular redox status differently, and their relations can have varying effects on cell viability and ferroptosis sensitivity.

This review offers an updated exploration of the role of the key GSH-related enzymes GPx4, Chac1 and GSTs in redox regulation of ferroptosis, focusing on both the regulation of the activity of each enzyme and their possible interactions, considering the impact on the risk of ferroptosis induction in cancer cells.

## 2. Ferroptosis: Basic Pathways

**Figure 1:**
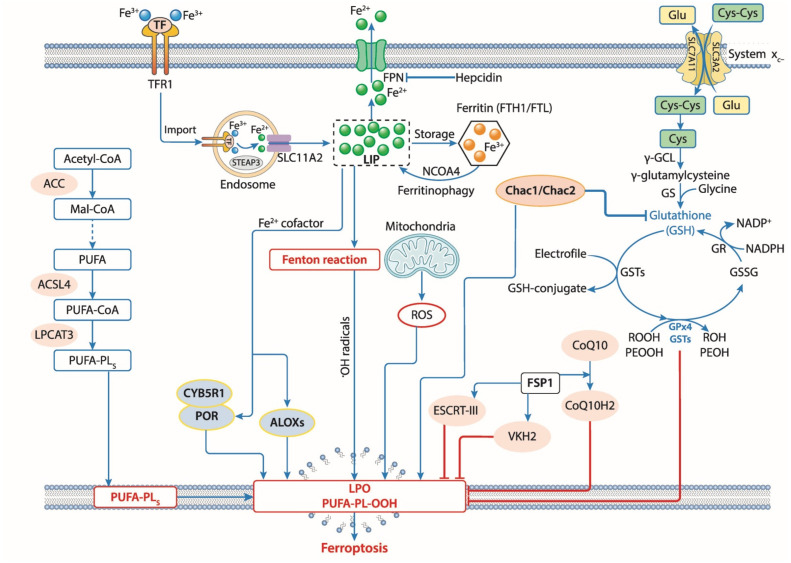
The core mechanisms of ferroptosis. Ferroptosis is a regulated cell death process characterized by iron accumulation, induction of lipid peroxidation (LPO), and an increase in lipid peroxides from polyunsaturated fatty acid-containing phospholipids (PUFA-PLs). PUFA-PL levels are regulated by PUFA synthesis, involving acetyl-CoA carboxylase (ACC), catalyzing the first reaction of fatty acid synthesis through carboxylation of acetyl-CoA to malonyl-CoA (Mal-CoA) and acyl-coenzyme A (CoA) synthetase long chain family member 4 (ACSL4), which converts polyunsaturated fatty acids (PUFAs), especially arachidonic acid, into acyl-CoA esters (PUFA-CoAs). Lysophosphatidylcholine acyltransferase 3 (LPCAT3) is a critical mediator of PUFA-PL synthesis and recovers PUFA-PLs through a reaction between lysoglycerolipids and PUFA-CoAs, influencing the level of phospholipid hydroperoxides and the risk of ferroptosis. Dietary iron (Fe^3+^) is transported by transferrin (TF) and enters cells via transferrin TF/transferrin receptor 1 (TFR1) endocytosis. Within endosomes, the metalloreductase STEAP3 reduces Fe^3+^ to Fe^2+^, forming the labile iron pool (LIP), which is transported by solute carrier family 11 member 2 (SLC11A2). LIP is balanced with Fe^3+^ storage in ferritin (FTH1/FTL subunits) and can be increased due to ferritinophagy activated by ferritin–nuclear receptor coactivator 4 (NCOA4). Cellular Fe^2+^ is exported by ferroportin (FPN), which is negatively controlled by hepcidin. Dysregulation of TF/TfR1 and STEAP3/SLC11A2 systems, along with increased hepcidin and NCOA4 levels, leads to intracellular iron accumulation and ferroptosis. Fe^2+^ ions activate LPO through the Fenton reaction, generating ^•^OH radicals that attack PUFA-PLs and act as cofactors for ALOXs and POR, further promoting LPO. NADH-cytochrome b5 reductase (CYB5R1) increases LPO through the generation of ROS. The main defense mechanisms against ferroptosis include the glutathione (GSH)-dependent system and the antioxidant system controlled by ferroptosis suppressor protein 1 (FSP1). The GSH system plays a crucial role in antioxidant defense against ROS, regulating the reduction of lipid peroxides and protecting cells from ferroptosis depending on the GSH level, which is maintained by its synthesis and recovery from oxidized glutathione (GSSG) by glutathione reductase (GR). GSH is synthesized from Glu, Gly, and Cys. Cys is the most essential and is reduced from cystine transported by the transmembrane cystine–glutamate antiporter (Xc− system or xCT), which consists of a light-chain subunit (SLC7A11) and a heavy-chain subunit (SLC3A2). In the first step of GSH synthesis, glutamate–cysteine ligase (GCL) catalyzes the reaction of Glu with Cys to form γ-glutamylcysteine, which reacts with Gly to form GSH, which is catalyzed by glutathione synthetase (GS). Glutathione peroxidases (GPxs, particularly GPX4) and glutathione S-transferases (GSTs) use GSH as a co-substrate for the reduction of hydroperoxides, predominantly phosphatidylethanolamine-OOHs (PE-OOHs), to alcohols (PE-OHs), effectively defending against increased oxidative stress and ferroptosis, whereas glutathione-specific gamma-glutamyl cyclotransferases (ChaC1/ChaC2) catalyze intracellular GSH degradation, which can lead to induction of ferroptosis. Ferroptosis suppressor protein 1 (FSP1) reduces CoQ10 (ubiquinone) to CoQ10H2 (ubiquinol) and vitamin K (VK) to VK hydroquinone (VKH2), which possess remarkable antioxidant properties, effectively trapping free radicals and inhibiting LPO and ferroptosis. FSP1 can also potentiate cell membrane repair and inhibit ferroptosis through the endosomal sorting complex required for the transport III (ESCRT-III)-dependent membrane repair pathway.

Ferroptosis is a form of programmed cell death that is defined as an iron-catalyzed form of regulated necrosis driven by iron-dependent phospholipid peroxidation, featuring the accumulation of ROS and LPO overproduction [21,25,26]. Ferroptosis may be determined as a consequence of a redox imbalance between its drivers and defense systems, including balance disruption between the production of phospholipid hydroperoxides and the activity of their GSH-dependent utilization, leading to activation of LPO, which can be triggered by growth of the Fe^2+^ pool (Figure 1). Increased levels of lipid peroxides, predominantly phosphatidylethanolamine-OOH (PE-OOH), result in ferroptosis, while iron seems to act as a catalyst regulator [39].

Among the polyunsaturated fatty acids (PUFAs), arachidonic acid (AA) and adrenoic acid (AdA) are the main substrates of membrane LPO in ferroptosis. The specific biomarker of ferroptosis, acyl-CoA synthetase long-chain family member 4 (ACSL4), is an enzyme involved in fatty acid metabolism that activates PUFAs into active acyl-CoA esters, controlling the PUFA content in phospholipids, which are susceptible to oxidation reactions leading to ferroptosis [40]. In addition to ACSL4, lysophosphatidylcholine acyltransferase 3 (LPCAT3) recovers polyunsaturated fatty acid-containing phospholipids (PUFA-PLs) through a reaction between lysoglycerolipids with AA-CoA, influencing the level of phospholipid hydroperoxides and the risk of ferroptosis [41]. It should be noted that other reactions of fatty acid synthesis or its metabolism can significantly contribute to increasing the risk of ferroptosis induction. For example, the activity of acetyl-CoA carboxylase (ACC) in catalyzing the first reaction of fatty acid synthesis and producing malonyl-CoA through carboxylation of acetyl-CoA is critical for PUFA synthesis and therefore necessary for ferroptosis [42].

In the initiation stage of LPO, ^•^OH radicals attack the double bonds and extract a hydrogen atom from PUFAs to produce carbon-centered phospholipid radicals (PL**˙**), which react with O_2_ to form phospholipid peroxyl radicals (PL-OO˙), causing free radical chain reactions and the production of large amounts of phospholipid hydroperoxides (PL-OOHs) and PL-OO˙, which are accelerated by ferrous ions [43,44]. ^•^OH radicals are produced not only by the Fenton reaction,

Fe^2+^ + H_2_O_2_ → Fe^3+^ + OH^−^ + ^•^OH,

but, in general, through the Haber–Weiss reaction, which includes the Fenton reaction

O_2_^•–^ + H_2_O_2_ → ^•^OH + OH^−^ + O_2_.

The peroxidation of PUFA-PLs is driven not only by non-enzymatic autoxidation initiated by the Fenton reaction needed in Fe^2+^ ions but also by the action of iron-dependent enzymatic reactions, primarily catalyzed by arachidonate lipoxygenases (ALOXs) and cytochrome P450 oxidoreductase [45]. Among ALOXs, which are enzymes containing non-heme iron and directly introducing oxygen to PUFAs and membrane PUFA-containing lipids, ALOXE3, ALOX5, ALOX12B, ALOX15, and ALOX15B are implicated in ferroptosis induction [46,47,48]. In addition, cytochrome P450 oxidoreductase (also known as POR, CYPOR, or CPR), a NADPH–hemoprotein oxidoreductase flavoprotein that transfers electrons from NADPH to all microsomal P450 enzymes, and NADH-cytochrome b5 reductase (CYB5R1), which is involved in the transfer of reducing equivalents from NADH due to the FAD electron acceptors in cytochrome b5, also accelerate ferroptosis [49,50]. POR and CYB5R1 can transfer electrons to molecular oxygen with the generation of H_2_O_2_, which causes the production of hydroxyl radicals (^•^OH) through the Fenton reaction with ferrous iron (Fe^2+^), leading to the peroxidation of PUFAs in membrane phospholipids and activation of ferroptosis. Genetic knockout of POR and CYB5R1 decreases cellular H_2_O_2_ generation, preventing lipid peroxidation and ferroptosis [49].

The main primary LPO products are lipid hydroperoxides, and the accumulation of PL-OOHs in cellular membranes is recognized as the hallmark and rate-limiting step of ferroptosis [26,51]. In contrast, among the secondary products of LPO, malondialdehyde (MDA) and 4-hydroxynonenal (4-HNE) are more connected with ferroptosis because of their capacity to form covalent adducts with biological macromolecules, crosslink and inactivate proteins, and reduce membrane integrity, thereby promoting cell membrane rupture and ferroptosis [52].

The high reactivity of MDA is based on its electrophilicity, which makes it strongly reactive toward nucleophiles, leading to the formation of adducts with proteins and DNA. It promotes intramolecular or intermolecular protein (i.e., on Lys, His, or Arg residues) and DNA crosslinking through Schiff base adducts that may induce profound alteration in the biochemical properties of biomolecules [53]. MDA is an important contributor to DNA damage and mutation to a major extent due to the formation of adducts to deoxyguanosine and deoxyadenosine [54]. 4-HNE’s high toxicity can be explained by its rapid reactions with thiols and amino groups because of its attachment to proteins via a Michael addition reaction (targeting Cys, His, and Lys residues) or through the formation of Schiff base adducts (on Arg and Lys residues), which leads to modulation of the activities of proteins [55,56]. Disruption in iron homeostasis, its overload, and consequent changes in cellular redox balance are the key drivers of ferroptosis [57,58]. Maintaining iron balance through coregulation of iron absorption, storage, and utilization is crucial to defend cells against ferroptosis. As a rule, in normal conditions, dietary iron, mainly as ferric iron (Fe^3+^), is transported by transferrin (TF) and enters cells through endocytosis using transferrin receptor 1 (TfR1). The labile iron pool (LIP) is formed by Fe^2+^ ions, which are transported by solute carrier family 11 member 2 (SLC11A2/DMT1) from endosomes, where the metalloreductase STEAP3 reduces Fe^3+^ to Fe^2+^ [59,60]. In addition, iron is stored in ferritin, which is composed of ferritin heavy-chain (FTH1) and ferritin light-chain (FTL) subunits, through chelating and oxidizing Fe^2+^ to the more stable Fe^3+^ for storage. Ferroportin (FPN, also known as SLC40A1) exports cellular iron (Fe^2+^) [61]. The process regulates cellular iron homeostasis through iron regulatory proteins (IRP1/2), which bind to iron-responsive elements (IREs) in target mRNAs. This post-transcriptionally controls the gene expression of iron metabolism proteins like ferritin, TfR1, SLC40A1, and DMT1 [62,63]. It should be noted that cytoplasmic iron is predominantly present as a complex of Fe^3+^ ions with GSH [64], which may be delivered to ferroportin via the cytoplasmic iron chaperone PCBP2 [65]. The flow of iron out of the cells is controlled by hepcidin through changing ferroportin conformation [66], as well as hepcidin-induced endocytosis and degradation of ferroportin [67].

Regulation of iron levels is supported through coordinated mechanisms, including the divalent metal transporter 1 (DMT1)–transferrin (Tf) system, the hepcidin–FPN axis, and the ferritin–nuclear receptor coactivator 4 (NCOA4) pathway [68]. Dysregulation of these pathways causes the disruption of iron metabolism and accelerated lipid peroxidation, leading to ferroptosis. The high expression of Tf and TFR1 in tumor cells is mainly to meet the iron requirement of tumor cell proliferation, and inducing ferroptosis in Tf and/or TFR1-overexpressing cancers is a potential cancer treatment strategy [69,70,71]. For example, in glioblastoma cells, ferroptosis induction by RSL3/FIN56 led to increased TFR1 expression and ROS generation, while the pro-ferroptotic effect of these drugs could be reversed by *TFR1* knockdown [72]. It should be noted that DMT1 is a major regulator of mitochondrial membrane potential, and its deficiency increases the activity of mitochondrial complex I and reduces that of complex III, leading to increased NAD+ production, which activates isocitrate dehydrogenase (IDH2) by promoting its deacetylation via sirtuin deacetylase 3 (SIRT3). This results in higher levels of NADPH and GSH, which improve antioxidant capacity during erastin-induced ferroptosis [73]. Increased hepcidin levels suppress FPN, leading to intracellular iron accumulation and inducing ferroptosis [74]. Conversely, translation downregulating hepcidin expression, as seen in the knockout of the key regulator of translation cytoplasmic polyadenylation element binding protein 4 (CPEB4), elevates ferroportin levels, reduces intracellular iron, and decreases lipid peroxidation, thereby modulating liver cancer progression by reducing ferroptosis sensitivity [75]. NCOA4 is a key cargo receptor that facilitates ferritinophagy by binding to FTH1, delivering ferritin to autophagosomes for degradation, and consequently increasing Fe^2+^ levels [76,77,78]. Elevated NCOA4 promotes ferroptosis; for instance, artesunate inhibits osteosarcoma growth by upregulating NCOA4, triggering ferritinophagy, increasing Fe^2+^ accumulation, and activating ferroptosis [79]. Conversely, disrupting the NCOA4-FTH1 interaction reduces available iron, thereby inhibiting ferroptosis [80].

Ferroptosis, as mentioned above, is the consequence of a redox imbalance between drivers and defense systems [21,25,26]. The main defense mechanisms against ferroptosis include the GSH-dependent system (discussed below), as well as ferroptosis suppressor protein 1 (FSP1) and coenzyme Q10 (CoQ10), which form the NAD(P)H/FSP1/CoQ10 axis [28].

Ferroptosis suppressor protein 1 (FSP1) has recently been discussed as an additional regulator of ferroptosis after GPx4 [28,81]. FSP1 is localized in the plasma membrane, endoplasmic reticulum, and Golgi, and N-myristylation is essential for its anti-ferroptotic function [82]. FSP1 (also known as apoptosis-inducing factor mitochondria-associated 2, AIFM2) is an isozyme of AIF, a group of flavoproteins, which is capable of initiating caspase-independent apoptosis [83]. FSP1 consumes NADH/NADPH as an NADH/NADPH-dependent CoQ10 oxidoreductase to reduce CoQ10 (also called ubiquinone) to CoQ10H2 (ubiquinol), acting as a lipophilic antioxidative scavenger of free radicals in the prevention of lipid peroxidation, as well as indirectly regenerating another antioxidant, α-tocopherol, to inhibit ferroptosis [84]. Due to its reductase activity with the use of NAD(P)H, FSP1 reduces vitamin K (VK) to VK hydroquinone (VKH2) in the redox-dependent cycle of naphthoquinone [85]. VKH2 possesses remarkable antioxidant properties, effectively trapping free radicals and inhibiting lipid peroxidation and ferroptosis through FSP1-mediated VK reduction [86,87,88].

It has been found that FSP1 can potentiate cell membrane repair and inhibit ferroptosis through the ESCRT-III-dependent membrane repair pathway [89]. The ESCRT (endosomal sorting complexes required for transport) machinery is a multi-subunit protein complex crucial for membrane remodeling and scission [90,91]. Among its five sub-complexes (ESCRT-0, ESCRT-I, ESCRT-II, ESCRT-III, and VPS4), ESCRT-III is key, with twelve subunits (CHMP1A, CHMP1B, CHMP2A, CHMP2B, CHMP3, CHMP4A, CHMP4B, CHMP4C, CHMP5, CHMP6, CHMP7, and IST1) forming higher-order multimers to facilitate membrane remodeling. ESCRT-III subunits CHMP5 and CHMP6 accumulate in the plasma membrane following ferroptosis activation. Knockdown of these subunits sensitizes human cancer cells (PANC1 and HepG2) to lipid peroxidation-mediated ferroptosis in vitro and in vivo, suggesting that ESCRT-III confers resistance to ferroptotic cell death, promoting cell survival under stress [92]. *FSP1* knockdown in cells suppressed RSL3-induced expression of CHMP5 and CHMP6 at the plasma membrane, whereas overexpression of CHMP5, a crucial subunit of the ESCRT-III-dependent membrane repair pathway, was shown to reverse ferroptosis induced by ferroptosis inducers and demonstrate the protective effect of FSP1 through the modulation of ESCRT-III [89].

FSP1 plays an important role in ferroptosis resistance in cancer cells, and its overexpression is closely associated with enhanced ferroptosis resistance. High expression of FSP1 was found in ferroptosis-resistant non-small-cell lung cancer and correlated with poor patient prognosis [93].

## 3. GSH-Related Enzymes in Redox Regulation of Ferroptosis

### 3.1. Key Enzymes of GSH Metabolism and Redox Control of Ferroptosis

GSH depletion or inhibition impairs antioxidant defenses, leading to toxic lipid hydroperoxide accumulation and potentially increasing ferroptosis risk [35,38,94,95]. GSH maintains cellular redox homeostasis through antioxidant protection, thiol–disulfide exchange, redox-dependent regulation of cell signaling and gene expression, detoxification of toxic compounds, and eicosanoid synthesis [35,38].

GSH can directly scavenge ROS (e.g., hydrogen peroxide, superoxide, and peroxynitrite) [12,13,14] and serves as a redox cofactor for GSH consumption enzymes, which are critical for cellular redox homeostasis, detoxification, and signaling, utilizing GSH by converting it to glutathione disulfide (GSSG) or conjugates [15]. The functional state of the GSH-dependent system is determined not only by activities of key GSH synthesis enzymes and transporters of GSH precursor amino acids, but also by the actions of GSH transporters and GSH-related enzymes.

Some GSH-related enzymes are key enzymes with antioxidant functions, such as GPxs, GSTs, and Grxs [18], whereas cytosolic ChaC1 and the membrane-bound enzyme GGT degrade GSH. ChaC1 specifically degrades intracellular GSH into cysteine–glycine and 5-oxoproline, controls the cellular redox state through GSH level regulation [96], and acts as a critical pro-ferroptotic component in cancer, causing oxidative stress and inducing iron-dependent lipid peroxidation [97].

In contrast to ChaC1, GGT hydrolyzes extracellular GSH into glutamate and cysteine–glycine, which can be cleaved by membrane-bound dipeptidases to glycine and cysteine and finally transported into the cell by neutral amino acid transporters, such as the alanine–serine–cysteine transport system (ASCT) [98]. GGT is a heterodimer consisting of a heavy and a light subunit, where the active site is located. The family of h13 GGT genes includes eight active subtypes, which are abnormally expressed in various types of cancers. For example, the *GGTLC2* gene encoding gamma-glutamyl transferase light is highly expressed in gastric cancer (GC) and may influence its occurrence, development, and liver metastasis by inhibiting ferroptosis. *GGTLC2* knockdown reduces GSH and GPX4 while increasing Fe^2+^, suggesting it may modulate ferroptosis via GSH metabolism [99]. It has been found that the main physiological role of hGGT is the hydrolysis of extracellular GSH, which is essential to maintaining cysteine levels in the body [100,101]. GGT can also hydrolyze extracellular GSSG and give cystine, which can be taken up by cells and is reduced to cysteine serving as a source for the de novo synthesis of GSH and proteins [102].

The biosynthesis of GSH occurs in the cytosol through a two-step pathway [35,103]. In the first step, glutamate–cysteine ligase (GCL), consisting of a GCL catalytic subunit (GCLC) and GCL modulatory subunit (GCLM), catalyzes the reaction of glutamate with cysteine to form gamma-glutamylcysteine (GGC). In the second step, glutathione synthetase (GS) catalyzes the reaction of GGC with glycine to form GSH. Low levels of cytoplasmic cysteine typically limit the rate of GSH synthesis.

Ferroptosis can be modulated through the regulation of GCL activity as a rate-limiting enzyme in GSH synthesis. Buthionine sulfoximine (BSO), the irreversible inhibitor of GCL, triggers ferroptosis in tumor cells, specifically hepatocellular carcinoma (HCC), leading to decreased GSH levels [104]. Enhanced expression of inositol-requiring enzyme 1 (IRE1α), which cleaves and downregulates the mRNA of GCLC, promotes sensitivity to ferroptosis in MDA-MB-231 human triple-negative breast cancer cells upon treatment with erastin [105]. Solamargine, the major steroidal alkaloid glycoside, induces ferroptosis in colorectal cancer (CRC) cells and xenografts through downregulated GSS and GPx4 [21].

It should be noted that GCLC contributes to ferroptosis resistance not only by synthesizing GSH but also through its non-canonical function by catalyzing γ-glutamyl-peptide synthesis, which limits the accumulation of glutamate, leading to protection against ferroptosis [106].

Cysteine, being the most essential for the rate of GSH synthesis, is formed mostly through transsulfuration of amino acids. It is easily oxidized to cystine, which becomes essential to maintain the cellular cysteine pool in cancer cells, and enters cells from the microenvironment via the sodium-independent transmembrane cystine–glutamate antiporter, which is known as the system Xc− or xCT [107] and is one of the crucial factors in the prevention/modulation of ferroptosis [108,109].

The system Xc− consists of a light-chain subunit (SLC7A11) and a heavy-chain subunit (SLC3A2). SLC7A11 predominantly functions as a transporter with high specificity for cystine and glutamate, while SLC3A2 primarily acts as a chaperone protein crucial for maintaining the stability of SLC7A11 [110]. The overexpression of SLC7A11 has been observed in multiple types of cancer, including prostate cancer, breast cancer, renal carcinoma, and colon cancer, and is closely associated with tumor initiation, progression, metastasis, and therapeutic resistance, contributing to tumor growth and survival by inhibiting ferroptosis through increasing GSH synthesis [108,111]. Inhibition of SLC7A11 activity and/or decreasing *SLC7A11* expression makes cells more vulnerable to ferroptosis due to decreased GSH levels and the buildup of lipid peroxides. SLC7A11 is transcriptionally regulated by activators, such as activating transcription factor 4 (ATF4), nuclear factor erythroid 2-related factor 2 (Nrf2), and the protein C-ets-1 (ETS1), and repressors including transcriptional factors such as BTB domain and CNC homolog 1 (BACH1), p53, activating transcription factor 3 (ATF3), and signal transducer and activator of transcription 1 (STAT1) during stress responses, such as oxidative stress, nutrient deprivation, endoplasmic reticulum stress, radiation, oncogenic stress, DNA damage, and immune stress, which modulate cellular sensitivity to ferroptosis [112].

MicroRNAs (miRNAs) and N6-methyladenosine (m6A) RNA modifications are critical post-transcriptional regulators of SLC7A11. For example, the N6-methyladenosine modification enhances ferroptosis resistance through inhibiting *SLC7A11* mRNA deadenylation in hepatoblastoma [113]. In contrast, miR-5096 and miR-375 promote ferroptosis in breast and gastric cancer cells, respectively, by targeting *SLC7A11* mRNA stability and/or translation [114]. The longevity of *SLC7A11* RNA can be supported by heterogeneous nuclear ribonucleoprotein C (HNRNPC), which takes part in the development of chemoresistance in CRC via reprogramming of ferroptosis pathways through shielding *Nrf2* and *SLC7A11* mRNAs from degradation [115]. The stability of SLC7A11 is controlled by deubiquitylase OTUB1 [116], which inhibits its degradation, enhancing ferroptosis resistance in cancer cells.

Inhibitors of the system Xc− and GSH depleters are included in the large group of small-molecular ferroptosis inductors (FINs) connected with repression of the SLC7A11–GSH axis in cellular antioxidant defense. The main inhibition strategies for this target include inhibition of cystine transport by SLC7A11 and cystine reduction by cytoplasmic thioredoxin reductase 1 (TXNRD1), inhibition of the GSH synthetic rate-limiting enzyme (GCL), and GSH depletion. Examples of these types of small-molecule ferroptosis inductors (FINs) are presented in Table 1.

Currently, the inclusion to FINs the inhibitors of glutathione reductase (GR) catalyzing the NADPH-dependent reduction of oxidized glutathione (GSSG) back to its reduced form is being intensively discussed because GR-mediated recycling of GSH is essential for Gpx4 activity in constraining ferroptosis [132]. Genetic or pharmacological suppression of GR accelerates ferroptosis, particularly in hypoxic or RAS mutant tumors [134]. Among 1,2,4-triazole–Schiff base succinate derivatives, AUR-517 has been reported as a potent GR inhibitor (IC50 = 0.471 ± 0.032 μM), while NQ-6 is the most effective in GR inhibition among naphthoquinones; this makes them prospective compounds for the development of ferroptosis-sensitizing agents with translational potential in oncology [132,133].

GPxs and GSTs are the most active in GSH consumption and use GSH as a co-substrate in the reduction of hydroperoxides to alcohols, effectively defending against increased oxidative stress and supporting cellular redox homeostasis, which limits ROS to a tumor-promoting level [16]. GPxs, particularly GPX4, are essential antioxidant enzymes with Se-dependent peroxidative function that protect cells from ferroptosis by neutralizing lipid peroxides. GPx4 is the leading enzyme protecting membranes from peroxidation damage through suppression of LPO products by reduction of phospholipid hydroperoxides, cholesterol hydroperoxides, and fatty acid hydroperoxides, acting as a primary endogenous inhibitor of ferroptosis [135]. Inhibiting GPx4 with the use of numerous FINs, often via GSH depletion (e.g., erastin) or direct inhibition (e.g., JKE-1674), triggers ferroptosis, providing a novel therapeutic strategy to combat treatment-resistant tumors [136].

In addition to detoxification through the conjugation reaction of GSH and reactive electrophiles generated by cytochrome P450 metabolism to form GSH conjugates, GSTs facilitate the detoxification process by catalyzing the conjugation of GSH with peroxides, thereby reducing cellular damage [137]. GSTs, particularly GSTP1, act as essential GSH-dependent antioxidant enzymes that inhibit ferroptosis by conjugating GSH with 4-HNE and reducing lipid hydroperoxides. While GPX4 is the primary regulator of ferroptosis, GSTP1 operates as an additional Se-independent defense mechanism [138]. GSTs possess not only catalytic activity but also the capacity for protein–protein interactions included in their role in regulating ferroptosis [139,140]. Through the NRF2/GSH/GST axis, GSTs protect cells from iron-dependent lipid peroxidation, with their dysfunction linked to cancer resistance and increased sensitivity to ferroptosis-inducing therapies [137,141].

The key roles of GhaC1, GPx4, and GSTs as GSH-related enzymes in the redox regulation of ferroptosis are discussed below.

### 3.2. ChaC1 Controls Oxidative Stress, Ferroptosis, and Cancer Drug Resistance

#### 3.2.1. ChaC1 and ChaC2: Structure and Functions

**Figure 2:**
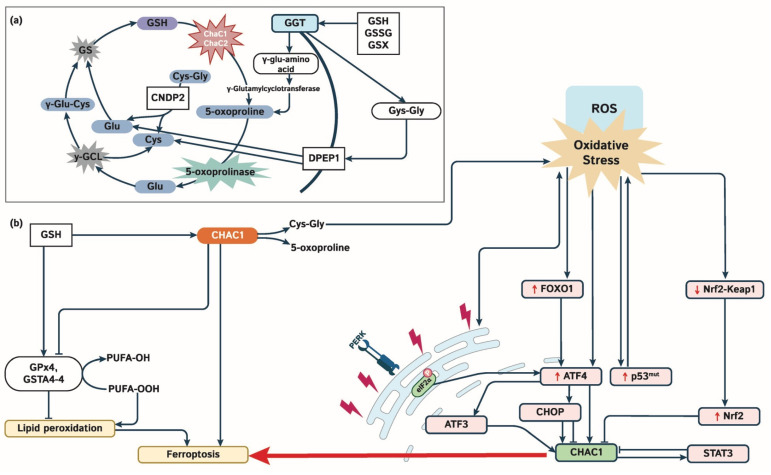
Role of ChaC1 in redox-dependent regulation of ferroptosis. (**a**) *ChaC1 and ChaC2 in GSH degradation and the γ-glutamyl cycle of GSH synthesis*. On the outer side of the cytoplasmic membrane, gamma-glutamyl transferase (GGT) hydrolyzes reduced and oxidized forms of glutathione (GSH, GSSG) and GSH conjugates (GS-X) and transfers a γ-glutamyl residue to the neutral amino acid, enabling its transport into the cell. ChaC1 and ChaC2 hydrolyze GSH to cysteinylglycine (Cys-Gly) and 5-oxoproline directly in the cell. Cys-Gly formed as a result of GGT, ChaC1, and ChaC2 is cleaved by dipeptidases (membrane-bound dipeptidase 1, DPEP1, and cytosolic carnosine dipeptidase 2, CNDP2) into cysteine (Cys) and glycine (Gly), which become the substrates for glutamate cysteine ligase (γGCL) and glutathione synthetase (GS). γ-Glutamylcyclotransferase breaks the bond between the γ-glutamyl residue and the amino acid, with the formation of a free amino acid and 5-oxoproline, which is decyclized by oxoprolinase with the formation of glutamic acid (Glu), which is also the substrate for γGCL. Cys and Glu are used to synthesize γ-glutamylcysteine (γ-Glu-Cys) by γGCL, and Gly is added to its C-terminus by GS to form GSH. (**b**) *ChaC1 in redox-dependent regulation of ferroptosis*. ChaC1 degrades GSH, which acts as a co-substrate for glutathione peroxidase 4 (GPx4) and glutathione transferase A4-4 (GST A4-4) for detoxification of lipid hydroperoxides (PUFA-OOHs) into non-toxic lipid alcohols (PUFA-OHs) and promotion of ferroptosis through increased ROS and lipid peroxidation. Some redox-dependent events are included in the transcriptional regulation of ChaC1. Oxidative stress and ER stress activate the PERK-eIF2α-ATF4 pathway, leading to the transcriptional upregulation of ChaC1 and subsequent GSH degradation. The transcription factors ATF4 and ATF3 bind to two critical cis-regulatory elements, ATF/cAMP and ATF/CRE, in the promoter of the *CHAC1* gene, enhancing its expression under ER stress conditions, and activation of the ATF4-CHOP-ChaC1 pathway may promote ferroptosis. The redox-dependent transcription factor FOXO1 also positively regulates ChaC1 through coordinated action with ATF4, while STAT3 negatively regulates *CHAC1* expression, but *CHAC1* overexpression can increase STAT3 phosphorylation. ChaC1-induced GSH depletion activates the NRF2 pathway, enhancing antioxidant defense mechanisms. While ChaC1-induced GSH depletion promotes Nrf2 activation, Nrf2 can inhibit *CHAC1* expression to prevent excessive GSH depletion, showing a fine-tuned control mechanism. ChaC1-induced oxidative stress can cause mutations in the *TP53* gene and the appearance of mutant p53 isoforms, which rather increase intracellular ROS.

One recently identified marker of ferroptosis is ChaC1 (cation transport regulator homolog-1 or glutathione-specific gamma-glutamyl cyclotransferase 1) [97,142,143]. ChaC1 is a cytosolic enzyme that functions as a γ-glutamyl cyclotransferase and specifically degrades GSH into cysteine–glycine and 5-oxoproline, controlling the cellular redox state through GSH level regulation (Figure 2a). As ChaC1 acts only on GSH (reduced glutathione) and not GSSG (oxidized glutathione), its action leads to an increase in the oxidizing environment of the cell [144]. Depending on cell types and/or its relations with cellular redox signaling, ChaC1 can promote cell death by reducing intracellular GSH levels and contribute to cancer progression due to its interactions with stress-related cellular mechanisms—especially through regulation of cell death pathways including ferroptosis [36].

The *CHAC1* gene is located on chromosome 15q15.1. ChaC1 has three isoforms, which vary in their structure and functionality. Isoform A (222 amino acid residues, 25 kDa), which is considered the canonical form, plays a key role in GSH breakdown and maintaining redox balance, while isoform B (shorter by 44 amino acids) is inactive and isoform X1 (264 amino acid residues) is not found in some higher species, including murine, elephant, canine, or chicken species [97]. ChaC1 is an inducible enzyme and can be expressed under specific stresses or pathological conditions, whereas its homologous counterpart ChaC2 (184 amino acid residues, 20.9 kDa), which localizes on chromosome 2p16.2, is constitutively expressed and exhibits catalytic efficiency for GSH 10–20 times weaker than that of ChaC1 [145]. Human ChaC1 has a Kcat of 225.2 min^−1^ for GSH (compared to the Kcat of 15.9 min^−1^ for ChaC2) and is localized in the cytoplasm rather than in the ER, Golgi, mitochondria, or nuclear compartments, and its localization remains unchanged after induction of the UPR (unfolded protein response) [146]. In contrast to GGT, which catalyzes extracellular degradation of GSH and provides an additional cysteine source for intracellular GSH synthesis, ChaC1 and ChaC2 play a key role in downregulating intracellular GSH levels, exerting a counteractive effect to regulate intracellular GSH homeostasis [36].

ChaC2 takes part in the regulation of the cellular redox state, and it plays an essential role in normal cell development through modulation of GSH maintenance. The reduction in ChaC2 expression leads to disruption of the cell cycle and cell death, supporting its importance in early developmental processes [147]. It is suggested that ChaC2 plays a dual role in cancer, in a context-specific manner [36]. ChaC2 acts as a tumor suppressor in such cancer types as gastric and colorectal cancers, where it is degraded by the ubiquitin–proteasome pathway, leading to inhibition of tumor cell growth and promotion of apoptosis and autophagy through ER stress pathways [148]. However, in breast cancer and lung adenocarcinoma, the overexpression of ChaC2 promotes tumor progression through an increase in ROS generation by reducing GSH and activation of the MAPK signaling pathway [149,150]. ChaC1 also plays a dual role in cancer, but its effects have a greater extent in contrast to ChaC2, as the inducible enzyme ChaC1 is included not only in the regulation of oxidative and ER stress and modulation of programmed cell death [151], but also in the control of cell drug resistance [152,153]. Low *CHAC1* expression serves as a key factor conferring tumor cell chemoresistance, while *CHAC1* overexpression can potentiate cell death through multiple mechanisms: activation of apoptotic pathways, degradation of ribonucleotide reductase, induction of autophagy and ferroptosis, activation of ROS generation, elevation of intracellular calcium levels, and loss of mitochondrial membrane potential [154,155].

Its action, through promotion of ferroptosis and apoptosis as well as mediation of ER stress, makes Chac1 a promising biomarker and therapeutic target, especially for overcoming chemoresistance, though its high expression often correlates with poor prognosis in some cancers like breast and kidney cancer by promoting aggressiveness [156,157]. In various cancer contexts, aberrant *CHAC1* expression has important clinical implications. For example, in kidney renal clear cell carcinoma, upregulated ChaC1 correlates with increased malignancy and serves as an independent risk factor for poor prognosis [157]. In glioblastoma, temozolomide treatment elicits robust upregulation of ChaC1; through activation of the c-Jun N-terminal kinase pathway, it enhances caspase-mediated apoptosis and disrupts Notch3 signaling, thereby contributing to tumor cell death [154].

#### 3.2.2. Transcriptional and Post-Transcriptional Regulation of ChaC1 and Its Effect on Ferroptosis in Cancer

CHAC1 expression is regulated by some transcription factors including ATF4, ATF43, NRF2, and STAT3 (Figure 2b). The transcription factors ATF4 (activating transcription factor 4) and ATF3 (activating transcription factor 3) bind to two critical cis-regulatory elements, the ATF/cAMP response element (ATF/CRE) and ATF/CRE modifier (ACM) element in the promoter of the CHAC1 gene, enhancing its expression under ER stress conditions. ATF4 binds primarily to the ATF/CRE and the ACM sites, especially under stress conditions, while ATF3 binds to the ATF/CRE site in both basal and stress conditions [158,159]. ATF4 is included in the PERK/eIF2α/ATF4 axis, which plays an essential role in activating UPR pathways and includes the induction of protein kinase RNA-like endoplasmic reticulum kinase (PERK), causing markedly elevated phosphorylation of Eukaryotic Initiation Factor 2 alpha (eIF2α) and promotion of a pro-adaptive signaling pathway by the inhibition of global protein synthesis and selective translation of ATF4. ChaC1 plays a crucial role in the UPR, and it is upregulated and primarily influenced by the transcription factor ATF4, and acts downstream of ATF3 and CHOP (DNA-damage-inducible transcript/CCAAT/enhancer-binding protein) [160]. ATF4 and ATF3 are important for control of CHAC1 expression, which is necessary for maintaining redox balance. ChaC1 can be induced by ATF4 under stress conditions such as amino acid starvation or oxidative stress. Activation of the ATF4-CHOP-ChaC1 pathway may promote ferroptosis. Thus, it was shown that artesunate induced ferroptosis in Burkitt’s lymphoma CA46 and Daudi cells, resulting in an endoplasmic reticulum stress response, by activation of the ATF4-CHOP-ChaC1 pathway [161]. The ATF3/ATF4-ChaC1 signaling pathway may be implicated as the central player in GSH degradation for overcoming cancer drug resistance that decreases chemotherapy efficiency. Overcoming bortezomib resistance in multiple myeloma, characterized by high proteasome activity, is a major challenge. Docosahexaenoic acid (DHA) or eicosapentaenoic acid (EPA) pretreatment significantly enhances bortezomib chemosensitivity in multiple myeloma cells [162]. This occurs by substantially reducing cellular GSH levels and altering the expression of the key enzymes in GSH metabolism. This effect is mediated by the activation of the Nrf2-ATF3/ATF4-ChaC1 signaling pathway and ferroptosis, highlighting the crucial role of GSH degradation in overcoming bortezomib resistance [162,163].

ATF4, as an integrated stress response effector (induced by oxidative stress, hypoxia, lactate accumulation, viral infection, heme depletion, etc.), controls the expression of a large set of genes involved in amino acid uptake or synthesis and upregulates the gene transcription not only of CHAC1 but also NFE2L2 (commonly NRF2) [164]. The transcription factor Nrf2 (nuclear factor erythroid 2-related factor 2) is activated upon oxidative and electrophilic stress and serves as an adaptive response program associated with redox regulation, metabolism, tumor therapy resistance, and immune suppression. Keap1 (Kelch-like ECH-associated protein 1) binds to Nrf2 in the cytosol and serves as an adapter between Nrf2 and the CUL3/RBX ubiquitin ligase complex, leading to the proteasomal degradation of Nrf2 [165]. Under conditions of oxidative or electrophilic stress, Keap1 is modified at several cysteine residues, weakening its interaction with Nrf2 and finally resulting in nuclear accumulation of Nrf2, which induces the transcription of genes containing an antioxidant response element (ARE). Transcriptional upregulation of the NFE2L2 gene and ChaC1-mediated GSH depletion have been identified as two independent mechanisms by which ATF4 exerts its NRF2-stimulatory function [164]. The upregulation of ChaC1 by ATF4 triggers GSH degradation, inducing oxidative stress and Nrf2 activation. This creates a feedback loop where Nrf2 boosts the cell’s oxidative stress response by enhancing GSH maintenance through increased gene expression of GCL as a rate-limiting enzyme in GSH synthesis and the light-chain subunit SLC7A11 of the cystine transporter system Xc− [166,167]. This Nrf2 effect is supported by its induction of GPxs, which use GSH as a co-substrate, and the thioredoxin system (thioredoxins, thioredoxin reductases, and peroxiredoxins) [165,168], as well enzymes of the pentose phosphate pathway (such as glucose-6-phosphate dehydrogenase and 6-phosphogluconate dehydrogenase) [169], which are involved in providing the NADPH needed for the reduction of oxidized glutathione (GSSG) and oxidized thioredoxins [170]. It was found that brusatol, obtained from the dried ripe fruit of Brucea javanica, effectively induced ferroptosis by upregulating the expression of CHAC1 and decreasing the level of Nrf2 and SLC7A11 in T24 and 5637 human bladder cancer cell lines [171]. The use of ferrostatin-1 (a selective inhibitor of ferroptosis) rescued cell viability compromised by brusatol treatment. In vivo, treatment with brusatol significantly suppressed tumor growth in nude mice. These findings demonstrate a potential approach for treating cancer in clinical environments through opposite regulation of Nrf2 and ChaC1. The interaction between Chac1 and the transcription factor STAT3 (signal transducer and activator of transcription 3) is complex. STAT3 negatively regulates CHAC1 expression in colorectal cancer cells. Propofol treatment, which suppresses STAT3, results in the upregulation of CHAC1 and the induction of ferroptosis in the human colon adenocarcinoma cells SW480 [172]. On the other hand, CHAC1 overexpression can increase STAT3 phosphorylation. Thus, it has been indicated that overexpression of CHAC1 increased the phosphorylation of STAT3^Y705^ in the non-small-cell lung cancer cells A549, whereas knockdown of CHAC1 significantly reduced STAT3^Y705^ phosphorylation [173]. The selective STAT3 inhibitor 7a, which blocks STAT3^Y705^ phosphorylation, was co-administered with overexpressed CHAC1, resulting in marked suppression of GLUT1 (solute carrier family 2), ENO1 (enolase 1), and LDHA (lactate dehydrogenase A) protein expression. These results confirm that ChaC1’s regulation of glycolytic genes is mediated via STAT3^Y705^ phosphorylation.

The redox-dependent transcription factor FOXO1 (forkhead box protein O1), a member of the FOXO transcription factor family, plays a complex role in cancer development and progression and can be involved in cancer prevention, treatment, and chemo/radioresistance [174,175]. While FOXO1 is generally considered a tumor suppressor due to its ability to induce cell cycle arrest, apoptosis, and DNA repair, it can also promote cancer cell survival and metastasis in certain contexts. ROS can damage cells and are also involved in both tumor suppression and promotion, making the relationship between FOXO1 and ROS in cancer a delicate balancing act. Among FOXO1-target genes, *CHAC1* is interesting due to its inclusion in cellular redox regulation through GSH destruction. Activation of FOXO1 under muscle hypertrophy leads to upregulation of the transcriptional factors C/EBPδ (CCAAT/enhancer-binding protein delta) and ATF4, which then coordinate to enhance the transcription of target genes, including *CHAC1* [176], and as a result, this associates the regulation of cellular redox states and stress responses with proteolytic and catabolic processes in muscle degradation.

The transcription factor p53 (p53 or transformation-related protein 53, TRP53) is a potent tumor suppressor, and its loss of function is associated with the development and progression of cancer. The *TP53* tumor suppressor gene is the most frequently altered gene in tumors, and mutant p53 proteins can acquire oncogenic properties, referred to as gain of function [177,178]. Regulation of p53 functions has a ROS-dependent character. Under a low intensity of ROS (mild, transient, tolerable, and mitigable stresses), p53 plays an antioxidative role, reducing ROS to protect the cell from damage (pro-survival), while high ROS levels (severe, prolonged, detrimental, and unrelievable stresses) lead to p53-dependent further intensification in ROS generation, resulting in cell death [179]. Mutant p53 isoforms fail to exert antioxidant activities and rather increase intracellular ROS, driving pro-tumorigenic survival with signaling and metabolic rewiring, as well as the modulation of critical ROS-related transcription factors and antioxidant systems, which lead to ROS imbalance linked to tumor progression [180]. It has been demonstrated that Helicobacter pylori (H. pylori) infection, particularly cagA-positive strains, in gastric epithelial cells leads to the overexpression of the ChaC1 enzyme, which in turn reduces intracellular GSH, increases ROS production, and induces oxidative stress, ultimately causing somatic mutations in the *TP53* gene [181]. The research indicates how H. pylori infection can contribute to the carcinogenic process in gastric cells by manipulating *CHAC1* expression and its enzyme activity and decreasing *TP53*, a critical tumor suppressor gene.

*CHAC1* expression is also regulated post-transcriptionally by non-coding RNAs (ncRNAs) that can lead to effects on tumor development and promotion of drug resistance or sensitivity in various cancers through changes in GSH levels (Table 2). For example, ChaC1 protein was upregulated by knockdown of the long non-coding RNA (lncRNA) GDIL (GSH Degradation Inhibiting LncRNA) [152]. The upregulation of GDIL was detected in platinum-resistant colorectal cancer and ovarian cancer cells. GDIL drove acquired platinum resistance by inhibiting GSH degradation instead of increasing GSH synthesis through GDIL/XRN2/ChaC1 pathways, and high levels of GDIL were associated with poor survival and hyposensitivity to chemotherapy in patients. Furthermore, the combination of GDIL inhibition and platinum led to delayed resistance. This effect relates to GDIL’s capacity to reduce *CHAC1* mRNA stability as a scaffold to enhance specific interactions between *CHAC1* mRNA and XRN2 (5′-3′ exoribonuclease 2), which is responsible for degradation of the 5′ fragment of RNA after cleavage of the polyadenylated region, which facilitates the completion of transcription. It has been suggested that GDIL not only interacts with XRN2 but also promotes its re-localization from the nucleus to the cytoplasm, where XRN2 could meet *CHAC1* mRNA and further degrade it.

The microRNA (miRNA) miR-432-5p also diminishes GSH degradation by targeting *CHAC1* mRNA, consequently inhibiting ferroptosis in prostate cancer cells, thereby increasing resistance to docetaxel. In 22RV1 and PC-3 cells, miR-432-5p originating from cancer-associated fibroblast exosomes effectively inhibited erastin-induced lipid-ROS accumulation and ferroptosis due to a decrease in *CHAC1* overexpression and promotion of GSH levels, activating GPx4 to prevent lipid peroxide accumulation [153].

Thus, its action as a master of GSH degradation makes Chac1 a critical factor in the redox promotion of ferroptosis, apoptosis, and ER stress, as well as overcoming chemoresistance through suppression of GSH-dependent limitation of ROS and lipid peroxidation overproduction.

### 3.3. GPx4, Ferroptosis, and Overcoming Chemoresistance in Cancer Cells

GPx4 plays a crucial role in preventing ferroptosis by protecting cells from the oxidative damage of lipids [184,185,186]. Among GPx isoforms, which include eight members (GPx1–8) and reduce small organic hydroperoxides using GSH as a co-substrate, only GPx4 can reduce large complex lipid hydroperoxides and cholesterols, even when they are embedded in the biological membrane [184].

In the decomposition reaction catalyzed by GPx4, H_2_O_2_ or lipid hydroperoxides (ROOHs) are reduced by GSH to H_2_O or the corresponding alcohol (ROH), thereby inhibiting LPO:

2 GSH + ROOH → GSSG + ROH + H_2_O.

Oxidized glutathione is reduced by GR, completing the cycle:

GSSG + NADPH(H^+^) → 2 GSH + NADP^+^.

**Figure 3:**
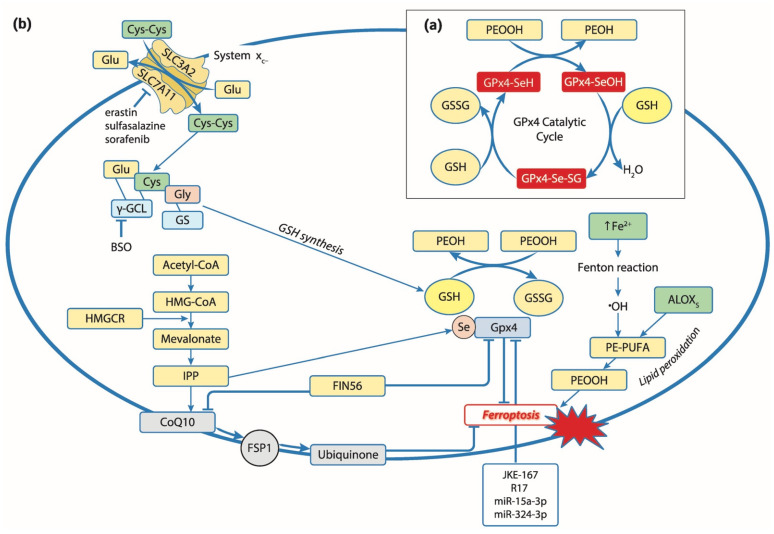
Role of GPx4 in the ferroptosis regulatory pathway. (**a**) *GPx4 catalytic cycle representation*. Under the reduction of lipid hydroperoxides, predominantly phosphatidylethanolamine-OOH (PE-OOH), to alcohols (PE-OHs), catalyzed by GPx4, GPx4 selenol (GPx4-SeH) is oxidized into selenic acid (GPx4-SeOH). Selenic acid is reduced to its active form, selenol, by a two-step reaction with GSH: the first GSH reacts with selenic acid to form a Se-glutathione intermediate (GPx4-Se-SG), which is reduced by the second GSH to selenol. (**b**) *Regulation of ferroptosis by GPx4*. The conversion of polyunsaturated fatty acids (PUFAs) of phospholipids to peroxide (PE-PUFA) represents the initiation step to drive ferroptotic cell death. The phosphatidylethanolamine hydroperoxides (PE-OOHs) are formed by non-enzymatic and enzymatic lipid peroxidation. The increased cytosolic labile iron pool (Fe^2+^ ions) activates the Fenton reaction and generation of ^•^OH radicals that initiate non-enzymatic LPO, which can be increased by arachidonate lipoxygenases (ALOXs). GPx4 converts toxic PE-OOH into non-toxic alcohol, thereby preventing ferroptosis. GPx4’s co-substrate, GSH, is synthesized from glycine, glutamate, and cysteine by the ATP-dependent enzymes GCL and GSS. The amino acid antiporter system Xc− (composed of SLC3A2 and SLC7A11 subunits) exchanges intracellular glutamate for extracellular cystine, which then converts to cysteine, contributing to GSH synthesis. Inhibition of the key enzymes of GSH synthesis—GCL (buthionine sulfoximine, BSO) and SLC7A11 (erastin, sulfasalazine, and sorafenib)—leads to activation of ferroptosis through GSH depletion. A selenocysteine residue is added to the catalytic center of GPx4 with the use of isopentenyl pyrophosphate (IPP), leading to GPX4 activation and ferroptosis inhibition. IPP is generated by the mevalonate pathway: acetyl-CoA is converted to 3-hydroxy-3-methylglutaryl-CoA (HMG-CoA). HMG-CoA is reduced by 3-hydroxy-3-methylglutaryl-CoA reductase (HMGCR) to mevalonate, which, in turn, is converted to IPP, generating coenzyme Q10 (ubiquinone, CoQ10), which is reduced to ubiquinol by ferroptosis-suppressor-protein 1 (FSP1) and leads to blocking of lipid peroxidation. The ferroptotic inducer FIN56 works through a dual mechanism of depleting the GPx4 protein and CoQ10 levels. Direct and indirect inhibitors of GPx4 (JKE-167 and R17) and non-coding RNAs (miR-15a-3p and miR-324-3p) targeting *GPX4* mRNA affect its enzyme activity and disrupt cellular redox balance, inducing oxidative stress and ferroptosis.

GPx4’s catalytic action is based on oxidation/reduction steps and involves the redox shuttling of the selenocysteine active site between an oxidized and a reduced state [135]. In the first phase, the reduction of lipid peroxides to non-toxic lipid alcohols occurs due to the oxidation of the active site selenol (Se-H) to selenic acid (Se-OH) (Figure 3a). During the second phase, the co-substrate GSH is used to reduce the selenenic acid back to active selenol and allow the oxidation/reduction process to be repeated. One molecule of GSH reacts with selenic acid to form a selenium–glutathione intermediate, while the second GSH reduces the Se-glutathione intermediate into selenol and releases GSSG, which is reduced by GR. GPx4 is considered the core regulator of ferroptosis since it reduces phospholipid hydroperoxides by catalyzing the conversion from ROOH into ROH, preventing iron-dependent lipid reactive oxygen production and inhibiting ferroptosis (Figure 3b) [184,187].

GPx4 is a monomer composed of a thioredoxin motif of four solvent-exposed alpha helices and seven beta strands, and it is present as three physiological isoforms: cytosolic (cGPx4), mitochondrial (mGPx4), and nuclear (nGPx4). *GPX4* can be expressed in a variety of tissues, with the highest content in the testis affecting the development and function of sperm [188]. It should be noted that cGPx4 is expressed in most mammalian cells, whereas mGPx4 mostly appears in spermatoid cells, and nGPx4 is expressed in late spermatocytes [189]. The isoforms cGPx4 and mGPx4 may independently inhibit ferroptosis, while the potential role of nuclear GPx4 requires further investigation. Notably, overexpression of mGPX4, but not cGPx4 or FSP1, can rescue mitochondrial LPO and ferroptotic cell death induced by RAS-selective lethal 3 (RSL3), which directly binds to and inactivates GPx4 in DHODH KO cells (with knockout of dihydroorotate dehydrogenase (DHODH)) [31].

Multiple strategies have been developed to inhibit GPx4 activity or promote its degradation to induce ferroptosis [186]. Decreased GSH maintenance indirectly affects GPx4 activity as its co-substrate and disrupts cellular redox balance, inducing oxidative stress and ferroptosis. Defects in the system Xc− are the most common reasons leading to suppression of GSH synthesis. Therefore, modulation of the system Xc−/GSH/GPX4 axis, which includes critical components of the antioxidant system, plays a key role in regulating LPO-mediated ferroptosis. As ferroptosis effectively inhibits tumorigenesis and eliminates cancer cells with high proliferative capacity, the methods of its activation, especially through the system Xc−/GSH/GPX4 axis, are actively promoted [19,190]. The strategy of modulating ferroptosis is a prospective treatment approach for overcoming cancer chemoresistance, especially for mesenchymal and dedifferentiated cancer cells, which typically exhibit resistance to apoptosis and traditional treatment approaches and display a remarkable susceptibility to ferroptosis [19].

GPx4 has been reported to be associated with tumor resistance, so suppression of its expression, mRNA silencing, and direct inhibition of enzyme activity might be used to overcome cancer chemoresistance through the induction of ferroptosis [19,190]. It was found that isoorientin induces cellular ferroptosis and reverses the resistance of lung carcinoma cells A549 to cisplatin by modulating the SIRT6/Nrf2/GPX4 signaling pathway, particularly through the downregulation of *GPX4* expression [191]. The use of *GPX4* siRNA leads to the downregulation of GPx4, induction of ferroptosis, and reversion of the oxaliplatin resistance in the human colorectal adenocarcinoma Caco-2 and HT-29 cell lines [192].

GPx4 is a challenging therapeutic target for small molecules because it lacks a traditional, deep “drug-like” binding pocket, possessing instead a shallow active site optimized for broad substrate interaction. Only a few GPx4 inhibitors are known, including ML162, ML210, JKE-167, and diarylfuroxan, and most of them bind to the active site of GPx4 through covalent interactions with the Sec46 residue [193,194]. These compounds induce ferroptosis; however, the application of these inhibitors is limited by their low selectivity and is pharmacokinetically unfavorable. It should be noted that the ferroptosis-inducing compounds RSL3 and ML162, as was currently found, completely lack the capacity to inhibit the enzymatic activity of the recombinant selenoprotein GPx4, but these compounds effectively inhibit cytosolic TXNRD1, which mediates the reduction of cystine to cysteine [195].

The new strategy of targeted protein degradation technology using proteolysis-targeting chimeras (PROTACs) has greater potential in targeting GPx4 [196,197,198]. A PROTAC is a heterobifunctional molecular degrader containing a target protein ligand, linker, and E3 ubiquitin ligase ligand. The target protein ligand specifically binds to the protein of interest (POI), whereas the E3 ligase ligand recruits the E3 ubiquitin ligase to form the POI-PROTAC-E3 ligase ternary complex, which promotes the ubiquitination of the target protein and finally degrades it via the proteasome. Unlike conventional small-molecule inhibitors, PROTACs degrade POIs catalytically, offering high selectivity and overcoming drug resistance [199,200].

Some prospective inhibitors have been produced in studies on GPx4-PROTAC’s recruitment of cereblon (CRBN, the substrate receptor of the CRL4^CRBN^ E3 ubiquitin ligase) and von Hippel–Lindau (VHL) E3 ligases [196]. For example, one of the new RSL3-based GPx4 degraders, compound R17, composed of a lenalidomide E3 ligand and a carbon chain linker (1 piperazine group together with 13 methylene groups), strongly reduced GPx4 protein expression in a dose-dependent manner, resulting in a half-maximal degradation concentration (DC50 37.4 nM, 6 h) for HT1080 fibrosarcoma cells and also exhibited potent GPx4 degradation ability in wild and gefitinib-resistant H1650 small-cell lung carcinoma cells (DC50 = 17.9 and 22.9 nM, correspondingly) [201].

Indirect inhibitors of GPx4 decrease the enzyme activity and induce ferroptosis by depleting GSH and inhibiting its synthesis through suppression of GCS as the rate-limiting enzyme in GSH synthesis (i.e., buthionine sulfoximine, BSO) and the system Xc− (i.e., erastin, sorafenib, and sulfasalazine), transporting the necessary GSH precursor cystine. Key examples include erastin, which inhibits cystine uptake, and HMGCR inhibitors (statins), which inhibit the mevalonate pathway essential for the synthesis of Sec-tRNA that is vital for Sec maturation, thus indirectly preventing the proper biosynthesis of GPx4 [202]. These agents disrupt the cellular antioxidant defense system, leading to activation of LPO and ferroptosis induction.

However, long-term maintenance with GPx4 inhibitors in vitro leads to altered metastatic profiles in vivo. As such, tumors derived from breast cancer cell lines with resistance to RSL3 and ML210 had unique metabolic and lipidomic profiles associated with decreased spontaneous metastases from primary tumors [203]. These results call for more careful consideration of the long-term use of GPx4 inhibitors.

Some chemotherapeutic drugs (i.e., cisplatin and altretamine) are able to promote ferroptosis through direct and indirect inhibition of GPx4. Cisplatin leads to GSH depletion and GPx4 inactivation, inducing both ferroptosis and apoptosis in A549 non-small-cell lung cancer cells and HCT116 colorectal carcinoma cells [204]. Altretamine (hexamethylmelamine) inhibits GPx4 and effectively kills U-2932 diffuse large B-cell lymphoma (DLBCL) cells in vitro [205]. A specific inducer of ferroptosis termed Ferroptosis Inducer 56 (FIN56) was identified as working through a dual mechanism of depleting GPx4 protein and mevalonate-pathway-derived coenzyme Q10 (CoQ10) [39].

NcRNAs, including miRNAs, lncRNAs, and circular RNAs (circRNAs), play significant roles in the modulation of GPx4 activity and function of the GSH-GPx4 axis, thus influencing cellular susceptibility to ferroptosis and overcoming drug resistance [206,207]. It was found that miRNAs can cause direct and indirect effects on GPx4 activity and regulate ferroptosis. MiR-15a-3p plays a regulatory role in various kinds of cancers and can also activate ferroptosis through targeting *GPX4* mRNA and inhibiting its enzyme activity in HCT116 colorectal carcinoma cells, leading to elevated intracellular levels of ROS and inducing LPO in the presence of erastin [208]. MiR-324-3p also directly targets and suppresses GPX4 expression, and its overexpression reverses the cells’ cisplatin resistance in A549 lung adenocarcinoma cells. The use of RSL3 mimics the effects of miR-324-3p upregulation. Therefore, the miR-324-3p/GPX4 cascade represents a promising target for enhancing cisplatin sensitivity in A549 cells [209]. Transfection of LNCaP prostate cancer cells with miR-15a also leads to a decrease in GPx4 protein expression, caused by the capacity of miR-15a to bind to the 3′-UTR region of *GPX4* mRNA, inducing ferroptosis and providing evidence for investigating the use of miRNA in therapeutic strategies against prostate cancer [210].

In contrast to miRNAs, lncRNAs exert an indirect influence on GPx4 through interactions with various molecules, such as miRNAs and transcription factors, thereby regulating gene expression and cellular processes. Thus, the lncRNA PAX8-AS1 can drive chemoresistance to gemcitabine and cisplatin in intrahepatic cholangiocarcinoma by activating NRF2-mediated *GPX4* transcription and stabilizing *GPX4* mRNA via insulin-like growth factor 2 mRNA-binding protein 3 (IGF2BP3) [211]. Targeting the PAX8-AS1/GPX4 axis with the GPx4 inhibitor JKE-1674 enhances the efficacy of these chemotherapeutic agents in preclinical models, offering a promising strategy to overcome chemotherapy resistance in intrahepatic cholangiocarcinoma. Ferroptosis in liver cancer cells can be induced through the lncPVT1/miR-214-3p/GPX4 axis [212]. LncPVT1 directly interacts with miR-214-3p to impede its role, acting as a sponge. Depletion of lncPVT1 by anesthetic ketamine accelerated ferroptosis in HepG2 and Huh7 cells due to decreased expression of GPX4, while ketamine-induced cell growth suppression and ferroptosis were also suppressed by miR-214-3p inhibition and GPX4 overexpression.

It has been found that long intergenic non-protein-coding RNA958 (LINC00958) was overexpressed in MDA-MB-157, MDA-MB-468, and MDA-MB-231 breast cancer cells, and its downregulation inhibited proliferation and promoted ferroptosis. This might be explained by LINC00958’s capacity to directly bind to serine/arginine splicing factor 1 (SRSF1), increasing the occupancy of SRSF1 on *GPX4* mRNA and augmenting its stability, thereby inhibiting ferroptosis of breast cancer cells [213].

CircRNAs are abnormally expressed in various cancers, acting as either cancer promoters or suppressors. Some circRNAs significantly regulate ferroptosis by modulating GPx4 expression. CircRNAs can function as microRNA sponges, RNA-binding protein (RBP)-binding molecules, transcriptional regulators, or protein translation templates. The interactions between circRNAs and RBPs are particularly important for regulating downstream gene expression [214]. For instance, in renal cell carcinoma (RCC) cells, high expression of circASAP1 interacts with HNRNPC (heterogeneous nuclear ribonucleoprotein C), an RBP involved in tumor growth regulation. This interaction leads to circASAP1 regulating GPx4 protein levels in RCC cells via HNRNPC, which reduces *GPX4* mRNA stability and subsequently affects its protein levels. Knocking down circASAP1 decreases GPx4 protein levels, which are crucial in ferroptosis [215].

CircRNA, such as the highly expressed circ_0082374 in NSCLC, can indirectly regulate gene expression by sponging miRNAs [216]. Silencing circ_0082374 inhibits NSCLC cell proliferation and metastasis while promoting ferroptosis. This is connected to the function of circ_0082374 to indirectly upregulate *GPX4* expression via sponging miR-491-5p, which reduces *GPX4* expression, and indicates the circ_0082374/miR-491-5p/GPX4 competitive endogenous RNA (ceRNA) network, thus highlighting a new molecular therapeutic target for NSCLC [217].

Some signaling mechanisms are included in the regulation of *GPX4* expression and modulation of ferroptosis in cancer cells. For example, upregulation of prostaglandin E receptor (PTGER3) weakens the epithelial–mesenchymal phenotype in triple-negative breast cancer and promotes ferroptosis both in vitro and in vivo by repressing *GPX4* expression [218]. On the other hand, downregulation of PTGER3 inhibits ferroptosis by increasing *GPX4* expression and activating the PI3K-AKT pathway. In esophageal squamous cell carcinoma (ESCC), *GPX4* expression was downregulated by knockdown of Aurora kinase A (AURKA), which led to activation of ferroptosis and suppression of cancer progression. AURKA acts as a tumor-promoting gene and may serve as a potential target for ESCC treatment [219]. Similarly, AURKA inhibition using siRNA or miR-4715-3p reconstitution in gastric cancer suppressed *GPX4* and induced cell death [220]. In glioblastoma multiforme cells, FOXO3 belonging to the forkhead box (FOX) family was found to upregulate the transcription of *GPX4* while also attenuating the degradation of *GPX4* mRNA through the linc00857/miR-1290 axis, thereby suppressing ferroptosis and promoting proliferation [221].

### 3.4. The Role of GST in Detoxification of Hydroperoxides and Regulation of Ferroptosis

**Figure 4:**
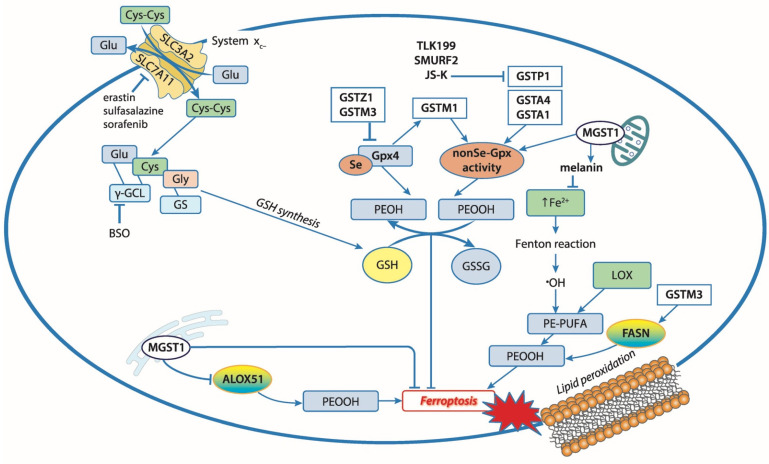
Multifaceted role of GSTs in the regulation of ferroptosis in cancer cells. GSTs (GSTP1, GSTA4, GSTA1, GSTM1, and MGST1) protect cells from oxidative stress and ferroptosis through detoxification of hydroperoxides (PE-OOHs) by selenium-independent glutathione peroxidase (nonSe-GPx) activity using GSH as a co-substrate, reducing lipid peroxides (PE-OOHs) to lipid alcohols (PE-OHs). The GSTP1 inhibitor TLK199, SMURF2-dependent GSTP1 ubiquitination and degradation, and *GSTP1* downregulation by JS-K through blocking the transcription factor c-Myc cause induction of ferroptosis. MGST1 can also suppress ferroptosis through binding to arachidonate 5-lipoxygenase (ALOX5), leading to reduced generation of PE-OOHs. In addition, MGST1 promotes melanin biosynthesis. Melanin binding with ferrous ions (Fe^2+^) decreases the yield of ^•^OH radicals by the Fenton reaction. GSTZ1 can act as a maleylacetoacetate isomerase, and its deficiency leads to succinylacetone accumulation and activation of the Nrf2 signaling pathway, increasing the GPx4 level, whereas GSTZ1 overexpression induces ferroptosis in cancer cells by Nrf2 and GPx4 downregulation. GPx4 stabilizes and post-translationally upregulates GSTM1 by protein–protein interactions. Upregulation of GPx4 and GSTM1 inhibits ferroptosis. GSTM3 acts not only by directly inhibiting GPX4 expression but also by stabilizing the ubiquitin-specific peptidase 14 (USP14)/fatty acid synthase (FASN) axis, leading to an increase in PE-OOH production. The catalytic activity of GSTs depends on the GSH level and GSH depletion through the inhibition of the key enzyme of GSH synthesis, GCL, by buthionine sulfoximine (BSO), as well as inhibition of the cystine–glutamate exchange antiporter, the system Xc− (SLC7A11) by erastin, and sulfasalazine, while sorafenib can lead to induction of oxidative stress and ferroptosis.

In addition to GPx, GST is another key enzyme of the GSH-dependent antioxidant system in the prevention of oxidative stress and ferroptosis through the detoxification of hydroperoxides using GSH as a co-substrate [222,223,224,225,226,227] (Figure 4). Three GST superfamilies are present in mammals: canonical soluble, mitochondrial, and membrane-associated enzymes. Cytosolic GSTs (cGSTs) are divided into alpha, mu, pi, omega, theta, sigma, and zeta (A, M, P, O, T, S, and Z) classes. Mitochondrial GSTs include the kappa (K) class (GST K1–1 is located in both mitochondria and peroxisomes) and GST isoforms of A, M, and P classes, which are synthesized in the cytoplasm and then transported to mitochondria. A novel superfamily designated MAPEG (membrane-associated proteins in eicosanoid and glutathione metabolism) consists of members of widespread origin with diversified biological functions and includes six human proteins: MGST1, MGST2, MGST3, 5-lipoxygenase-activating protein (FLAP), leukotriene C4 (LTC4) synthase, and microsomal prostaglandin E2 synthase 1 (MPGES1, earlier referred to as MGST1-L1) [222].

The members of GSTs are important phase II detoxification enzymes involved in the detoxification of exogenous and endogenous substances [228]. The hydrophilic GSH-conjugates (R-SG) formed intracellularly are excreted from the cell by the multidrug resistance-associated protein, MRP. Among the major types of GST-catalyzed reactions, including epoxide ring opening, nucleophilic aromatic substitution reactions, Michael addition of α, β-unsaturated aldehydes and ketones, and isomerization, peroxidase reactions play significant roles in cellular antioxidant defense [228,229]. In contrast to GPxs, GSTs have selenium-independent GPx activity and can reduce hydroperoxides of phospholipids and free fatty acids, as well as cholesterol hydroperoxides [222,223]:

2 GSH + ROOH → GSSG + ROH + H_2_O.

Alpha-class GSTs play an important role in the regulation of the intracellular concentrations of LPO products that may be involved in the signaling mechanisms of apoptosis. Both GSTA1 and GSTA2 can utilize fatty acid hydroperoxides and reduce PL-OOHs in biological membranes [230]. GSTA1 suppresses ferroptosis by exerting its peroxidase function to regulate resistance of HCC to sorafenib, which is a new multikinase inhibitor and exhibits a significant anticancer effect in the treatment of solid tumors [9]. Upregulation of GSTA1 expression is mediated by the transcription factor CTNNB1 (β-catenin), resulting in the formation of a cytoplasmic complex GSTA1-CTNNB1, which facilitates the nuclear translocation of CTNNB1. The combined use of GSTA1 and CTNNB1 inhibitors (curzerene and β-catenin-IN-2, correspondingly) demonstrated synergistic antitumor effects and overcame sorafenib resistance by targeting ferroptosis in cell xenograft models, organoids, and cells [231].

GSTA4 possesses a higher affinity for 4-HNE, which is a potentially toxic stable product of LPO, a common denominator in stress-mediated signaling and a pro-apoptotic second messenger altering cell cycle signaling pathways in a concentration-dependent manner [232]. 4-HNE is associated with different types of cell death, including not only apoptosis and necrosis but also autophagy, pyroptosis, necroptosis, parthanatos, oxeiptosis, cuproptosis, and ferroptosis, and has a significant role in the pathogenesis of acute and chronic diseases, especially degenerative and malignant diseases [233].

It has been found that 4-HNE is produced by Enterococcus faecalis-infected macrophages and mediates the microbiota-induced bystander effect (MIBE), leading to colorectal cancer [234]. Overexpression of GSTA4 is essential for protecting macrophages from E. faecalis-induced ferroptosis. In contrast, GSTA4 inactivation leads to activation of ferroptosis and blocking of MIBE by eliminating macrophages, thereby attenuating E. faecalis-induced colitis and CRC.

Expression of GSTP1 is highly increased in cancer. It was shown that the expression of GST isozymes is upregulated in 60 human tumor cell lines, both at the mRNA and protein levels, and GSTP1 is the most abundant isozyme in all of the cell lines [235]. Overexpression of *GSTP1* has been found in different tumors including pancreatic, breast, and lung tumors and may be involved in the formation of cancer cell resistance to chemotherapeutics, such as resistance of ovarian cancer cells against carboplatin and cisplatin, adriamycin-resistance of breast cancer cells and prostate cancer cells, resistance of gastric cancer cells against fluorouracil (5-FU) and cisplatin, and resistance of neurogliomas against cisplatin and irinotecan [228,236,237]. GSTP1 is a cytosolic enzyme that exerts peroxidase activity by directly converting 4-HNE and lipid hydroperoxides into non-toxic lipid alcohols in a selenium-independent manner and prevents cancer cells from ferroptosis death, induced, for example, by ionizing irradiation [140]. The use of the nitric oxide prodrug JS-K led to induction of ferroptosis in the human renal carcinoma cell (RCC) lines 786-O and A498, not only by reducing cellular GSH levels, increasing LPO, and elevating ferrous ion levels, but also by downregulation of GSTP1 through blocking the transcription factor c-Myc [238].

The photosensitizer aloe-emodin (AE) could induce cellular ferroptosis based on its specific inhibiting activity to GSTP1. AE@RBC/Fe nanocrystals (NCs), containing AE NC cores, modified red blood cell (RBC) membranes, and ferritin, exhibit significantly enhanced therapeutic effects in vitro and in vivo due to the two combined antitumor mechanisms provided by photodynamic therapy (PDT)/ferroptosis synergistic actions [239].

GSTP1 was identified as a direct target of miR-325-3p, and the miR-325-3p/GSTP1 axis is a new target for the regulation of paclitaxel resistance in triple-negative breast cancer (TNBC) by modulating ferroptosis [240]. MiR-325-3p was found to be downregulated in paclitaxel-resistant TNBC cell lines (MDA-MB-231/Pac and SUM159PT/Pac). Its overexpression increased paclitaxel sensitivity by promoting ferroptosis, whereas GSTP1 restoration partially reversed miR-325-3p-mediated ferroptosis and paclitaxel sensitization.

Downregulation of GSTP1 opens one more possibility for activating ferroptosis in cancer cells. SMAD-specific E3 ubiquitin protein ligase 2 (SMURF2) caused GSTP1 ubiquitination, leading to its degradation [138]. Genetic modulation of the SMURF2/GSTP1 axis or the pharmacological inhibition of GSTP1’s catalytic activity sensitized tumor responses to ferroptosis-inducing drugs both in vitro and in vivo. Thus, use of the GSTP1 inhibitor TLK199 improved the sensitivity of fibrosarcoma HT 1080 cells and xenografts’ responses to ferroptosis induced by sulfasalazine (SAS), inhibiting the system Xc−, while GSTP1 overexpression produced the opposite effect. By contrast, ectopic expression of SMURF2 was found to promote SAS-induced ferroptosis in a GSTP1-dependent manner.

GSTs possess not only catalytic activity but also the capacity for protein–protein interactions, which contribute to their role in regulating ferroptosis. It has been found that GPx4 stabilizes and post-translationally regulates the level of GSTM1 [241]. A significant reduction in the protein level of GSTM1 following the knockdown of *GPX4* was found in the brain metastatic lung cancer cells PC9-BrM3; conversely, the protein level of GSTM1 was upregulated following the overexpression of *GPX4* in the human lung cancer cells PC9. However, GPx4 did not cause changes in *GSTM1* expression or *GSTM1* mRNA levels. Upregulation of both GPx4 and GSTM1 inhibits ferroptosis induced by platinum drugs (cisplatin and carboplatin), leading to chemotherapeutic resistance.

In contrast, it has been revealed that under ionizing radiation-induced ferroptosis, the overexpression of GSTM3 reduced the expression of GPx4 in human nasopharyngeal carcinoma (NPC) cell lines (5-8F, HONE1, and CNE2) [242]. The interaction between GSTM3 and GPx4 in the cytoplasm was confirmed via Co-IP assay. Moreover, in the subcutaneous tumor xenograft model, it has been shown that in the promotion of radiation-induced ferroptosis, GSTM3 acted not only by directly inhibiting GPx4 expression but also by stabilizing the ubiquitin-specific peptidase 14 (USP14)/fatty acid synthase (FASN) axis, leading to an increase in lipid peroxide production because FASN is a key enzyme involved in lipid biosynthesis. USP14, a major deubiquitinase reversibly associated with the proteasome, participates in ionizing radiation-induced DNA double-strand break repair via non-homologous end joining [243], and GSTM3 causes suppression of the ubiquitination and subsequent degradation of FASN by stabilizing USP14.

The GSTM3 protein level can be decreased by Echinatin, a natural compound extracted from *Glycyrrhiza* plants, through promoting its proteasomal degradation, which leads to an increase in mitochondrial ROS production and induction of ferroptosis [244]. Echinatin triggered ferroptosis by activating GSTM3-mediated ROS/MAPK signaling (induction of phosphorylation of JNK and p38) and inhibiting GSTM3-mediated ferroptosis negative regulation proteins (the decrease in FTH1, Xc−, and GP4 protein levels) that led to suppression of the proliferation of cutaneous squamous cell carcinoma (cSCC) cells and tumor growth in vivo.

GSTZ1 not only displays detoxification activity like the other GST isoforms, but can also act as a maleylacetoacetate isomerase (MAAI), which catalyzes the isomerization of maleylacetoacetate to fumarylacetoacetate and is essential for phenylalanine metabolism [245]. GSTZ1 deficiency can lead to succinylacetone accumulation and activate the Nrf2 signaling pathway [246]. Low levels of *GSTZ1* expression were found in HCC cell lines (HepG2-SR and SUN449-SR) resistant to sorafenib. GSTZ1 depletion enhanced the activation of the Nrf2 pathway and increased GPx4 levels, thereby suppressing sorafenib-induced ferroptosis [247].

GSTZ1 induces ferroptosis in cancer cells not only by Nrf2 and GPx4 downregulation, but also through upregulating high mobility group box 1 (HMGB1) nuclear protein, which functions as a DNA-binding architectural chromatin factor regulating gene transcription, DNA repair, and stability [248,249]. GSTZ1 is significantly downregulated in BIU-87 bladder cancer cells. In contrast, in the study on GSTZ1 overexpression, downregulation of GPx4 and GSH was shown, greatly increasing the levels of iron, MDA, ROS, and transferrin, decreasing cell proliferation and activating HMGB1/GPX4 signaling. It has been concluded that GSTZ1 induces ferroptosis by activating the HMGB1/GPX4 axis in bladder cancer cells and that inhibition of this axis can antagonize the antitumor activity of GSTZ1 [248].

The E3 ubiquitin ligase UHRF1 (ubiquitin-like, containing PHD and RING finger domains, 1) is an epigenetic regulator coordinating DNA methylation and histone modifications, which can downregulate the expression of *GSTZ1* by modulating DNA methylation, thereby affecting ferroptosis in liver cancer cells [250].

It has been revealed that GSTM1 and GSTT1 protect renal tubular cells against cisplatin-induced nephrotoxicity and ferroptosis [251]. GSTT1 and GSTM1 were shown to be downregulated in the renal tubular cells of patients with acute kidney injury, caused by cisplatin treatment. The results suggest the need to genetically screen for GSTM1 and GSTT1 polymorphisms, which can help to determine a standard cisplatin dose for cancer patients undergoing chemotherapy.

Microsomal glutathione transferase 1 (MGST1) is a member of the MAPEG family [226,252,253,254] and an abundant membrane-bound enzyme with high levels of localization in the endoplasmic reticulum and outer mitochondrial membranes. It is a homotrimer that binds three molecules of GSH and possesses both GST and GPX activities, based on stabilizing GSH thiolate in the same active site and being specific toward multiple substrates, including products of lipid peroxidation, which are crucial for reducing lipid peroxidation and protecting membrane structures from oxidative stress.

MGST1 is overexpressed in various cancers, including pancreatic, glioma, gastric cancer, and melanoma [255,256,257,258], and plays a significant role in inhibiting ferroptosis as a redox-sensitive repressor, making MGST1 a potential therapeutic target [226]. MGST1 can suppress ferroptosis not only as a non-selenium-dependent GPx, but also through binding to ALOX5, leading to reduced generation of lipid peroxides [255]. In addition, a positive correlation was found between MGST1 expression and the production of melanin [259], which has a distinct protective effect on lipid peroxidation, binding ferrous ions and decreasing the yield of hydroxyl radicals [260].

High expression levels of MGST1 and mammalian rRNA N6-adenosine-methyltransferase (METTL5), a member of the conserved methyltransferase-like protein (METTL) family, are a prognostic risk factor for patients with HCC [261]. METTL5 regulates MGST1 protein expression via N6-methyladenosine catalytic function, increasing *MGST1* mRNA translational efficiency and MGST1 protein levels. This action directly contributes to cellular redox homeostasis and ferroptosis resistance in cancer cells. Thereby, the METTL5-MGST1 axis controls proliferation, migration, invasion, and suppression of ferroptosis in HCC cells.

*MGST1* overexpression has a significant role in the formation of cancer chemoresistance. For example, high MGST1 expression contributes to cisplatin resistance of non-small-cell lung cancer (NSCLC) cells by inhibiting ALOX5-induced ferroptosis [262]. The transcription factor zinc finger protein 384 (ZNF384) confers the resistance of glioma cells to temozolomide through inhibition of ferroptosis by positively regulating *MGST1* overexpression [38]. MGST1 is highly expressed in radiation-resistant non-small-cell lung cancer cells (NCI-1299-IR and HCC827-IR cells) and its knockdown epigenetically enhances radiotherapy cellular sensitivity by promoting ALOX15-mediated ferroptosis through regulation of the heme oxygenase-1 (HO-1)/DNA methyltransferase 1 (DNMT1) pathway [263].

## 4. Conclusions

Ferroptosis, as an iron-dependent, non-apoptotic form of regulated cell death, acts as an innate tumor suppressor mechanism and participates in the biological processes of tumors. As ferroptosis is caused as a consequence of a redox imbalance between its drivers and defense systems, disruption of the balance between the activation of LPO and production of phospholipid hydroperoxides on the one hand and the rate of their GSH-dependent utilization on the other may be one of the critical mechanisms of its redox regulation. The GSH system plays a crucial role in antioxidant defense against ROS, regulates the reduction of lipid peroxides, and protects cells from ferroptosis depending on the GSH level, which increases in different types of cancer, as does the expression of GSH-related enzymes, playing a significant role in the regulation of cellular redox homeostasis. Nevertheless, the GSH-related enzymes GPx4, Chac1, and GSTs have opposite effects on the regulation of ferroptosis. GPx4 and GSTs, as enzymes with Se-dependent and Se-independent peroxidase activities, serve in antioxidant protection, reducing hydroperoxides to alcohols, effectively defending cells against oxidative stress and ferroptosis, and thus supporting cellular redox homeostasis, whereas ChaC1/ChaC2 catalyze intracellular GSH degradation, which leads to the induction of ferroptosis, especially during excessive endoplasmic reticulum stress. Discussion of the mechanisms of these enzymes’ actions and influences on ferroptosis induction expands our understanding of the pathways and methods regulating and suppressing the vital activity of cancer cells. Triggering ferroptosis through the modulation of expression and activity of GPx4, GSTs, and ChaC1 might be a prospective pathway in the development of more targeted cancer treatments.

## Figures and Tables

**Table 1:** Inhibitors of the SLC7A11–GSH axis and induction of ferroptosis in cancer.

Effect on SLC7A11–GSH axis	Inhibitors	Cancer Type	Related Mechanism	Reference
Inhibition of SLC7A11 activity and cystine consumption	Erastin, sorafenib, sulfasalazine (SAS), tirapazamine (TPZ), pien-tze-huang (PZH), curcumenol, saikosaponin A, pseudolaric acid B (PAB), tanshinone IIA (Tan IIA)	Ovarian cancer, renal cell carcinoma (RCC), esophageal cancer, osteosarcoma, HCC, triple-negative breast cancer, glioma, and colorectal cancer	Erastin irreversibly inhibits system Xc−; sorafenib, SAS, TPZ, PZH, curcumenol, saikosaponin A, PAB, and Tan IIA downregulate the expression of SLC7A11	[117,118,119,120,121,122,123]
Inhibition of TXNRD1-mediated reduction of cystine to cysteine	Auranofin, PK11007, glaucocalyxin	Pancreatic ductal adenocarcinoma and non-small-cell lung cancer	Auranofin, PK11007, and glaucocalyxin irreversibly inhibit TXNRD1	[124,125,126]
Inhibition of GSH synthesis	Buthionine sulfoximine (BSO), glyphosate (GLY)	Kidney and ovarian cancer	BSO irreversibly inhibits GCL, the key enzyme in GSH synthesis; GLY inhibits GSH biosynthesis via blocking the phosphorylation and nuclear translocation of Nrf2	[127,128]
GSH depletion	Ponicidin, nanoparticles (NP-DNs), amphiphilic polymer prodrug of SO_2_, mPEG-PLG(DNs); nanovehicle of arginine–glycine–aspartate (RGD) modified mesoporous silica-coated iron oxide loading Fin56 (FSR-Fin56)	Pancreatic and gastric cancer	Ponicidin covalently binds to GSH, NP-DNs deplete GSH, and FSR-Fin56 depletes GSH and promotes GPX4 protein degradation	[129,130,131]
Inhibition of GSSG reduction	1,2,4-triazole–Schiff base succinate derivative AUR-517, naphthoquinone 6 (NQ-6)	Melanoma A375 cells and gastric cancer NUGC3 cells	AUR-517 and NQ-6 are potent GR inhibitors, forming stable interactions with GR key catalytic residues	[132,133]

**Table 2:** Some effects of non-coding RNAs on ChaC1 in cancer.

ncRNAs	Effect on GSH Maintenance	Cancer Type	Related Mechanism	Reference
lncRNA GDIL	Promotion of GSH level	Colorectal and ovarian cancers	High levels of GDIL are associated with poor survival and hyposensitivity to chemotherapy due to its action as a scaffold for *CHAC1* mRNA and XRN2 to promote platinum resistance in cancer	[152]
miR-432-5p	Promotion of GSH level	Prostate cancer	In 22RV1 and PC-3 cells, miR-432-5p effectively inhibits erastin-induced lipid-ROS accumulation and ferroptosis through the decrease in CHAC1 overexpression	[153]
CircLPAR3	Promotion of GSH level	Prostate cancer	CircLPAR3 is upregulated in docetaxel-resistant cells, promotes proliferation, and suppresses ferroptosis via the PCBP2/CHAC1 axis; m^6^A-modified circLPAR3 inhibits CHAC1 expression by competitively binding to RNA-binding protein PCBP2	[182]
Loc100506691	nd	Gastric cancer	Loc100506691 negatively regulates CHAC1 expression by modulating miR-26a-5p/miR-330-5p expression; the anti-proliferative effect of metformin in gastric cancer is partially caused by suppression of the Loc100506691-miR-26a-5p/miR-330-5p-CHAC1 axis	[183]

## Data Availability

No new data were created or analyzed in this study. Data sharing is not applicable to this article.
